# Innate Phagocyte Polarization in the Oral Cavity

**DOI:** 10.3389/fimmu.2021.768479

**Published:** 2022-01-06

**Authors:** Sarah Metcalfe, Natalie Anselmi, Alejandro Escobar, Michelle B. Visser, Jason G. Kay

**Affiliations:** ^1^ Department of Oral Biology, School of Dental Medicine, University at Buffalo, Buffalo, NY, United States; ^2^ Instituto de Investigación en Ciencias Odontológicas, Facultad de Odontología, Universidad de Chile, Santiago, Chile

**Keywords:** macrophage, neutrophil, inflammation, cellular polarization, periodontal disease, oral cancer

## Abstract

The oral cavity is a complex environment constantly exposed to antigens from food and the oral microbiota. Innate immune cells play an essential role in maintaining health and homeostasis in the oral environment. However, these cells also play a significant role in disease progression. This review will focus on two innate phagocytes in the oral cavity: macrophages and neutrophils, and examine their roles during homeostasis and disease development, with a focus on periodontal disease and cancer. Macrophages have a well-known ability to polarize and be activated towards a variety of phenotypes. Several studies have found that macrophages’ polarization changes can play an essential role in maintaining health in the oral cavity and contribute to disease. Recent data also finds that neutrophils display phenotypic heterogeneity in the oral cavity. In both cases, we focus on what is known about how these cellular changes alter these immune cells’ interactions with the oral microbiota, including how such changes can lead to worsening, rather than improving, disease states.

## Introduction

The oral cavity is the main gateway into the human body, leading to the respiratory and gastrointestinal tracts. It has a wide variety of microbial niches, and has the second most abundant microbiota after the gastrointestinal tract, consisting of ~800 bacterial species categorized into six major phyla (1). In this context, similarly to other mucosal barriers, the local oral immune system needs to find a balance of coexisting with the commensal microbiota while responding appropriately to pathogens (2). The crosstalk between microbiota and the innate immune system is essential to maintaining this host-microbe homeostasis, with the commensal microbiota itself playing a crucial role in regulating immune homeostasis (2–4). Indeed, the oral cavity is a unique mucosal environment where immune cells must be able to recognize and eliminate pathogens while maintaining tolerance to food antigens and the resident microbiota. Oral immunity is composed of a diverse and dynamic network of interactions with both innate and adaptive immunity components contributing to the maintenance, integrity, and host protection of oral tissues. However, as innate immunity is the first line of defense, it plays a pivotal role in both protecting the host and maintaining homeostasis (5, 6). While the saliva and gingival crevicular fluid (GCF) contain host immune molecules that can respond rapidly to protect the periodontium and the oral hard tissues against pathogens such as antimicrobial peptides, complement, and secretory IgA (7), these defense mechanisms only provide short-term protection and have a limited specificity (8). The oral mucosa resident and transmigrating immunologically active innate immune cells, including macrophages and neutrophils, also play important roles in maintaining effective immune surveillance.

The importance of innate professional phagocytes in maintaining a healthy and mature immune system is revealed upon a change in ‘ideal’ functional inflammatory immune cell infiltrate: such changes lead to degradation in the health of the periodontal tissues (4). For example, a lack of neutrophil infiltration into the oral cavity (neutropenia) leads to an increase in periodontal disease (9) while an overabundance and dysregulation of neutrophils during periodontal disease causes host tissue damage (10). Similarly, a reduction in macrophage numbers during aging contributes to an increase in periodontal disease (11), while macrophages themselves also contribute to the alveolar bone resorption seen during *P. gingivalis* induced periodontal disease (12). Recent data suggests part of this finely tuned balance of phagocytes is likely due to phenotypic variance within these cell types. Macrophages are responsible for not only host defense, but also have important tissue repair and homeostatic roles (13) and possess a spectrum of phenotypes with different responses during host and microbial interactions (14); (15). Neutrophil phenotypes during infection, inflammation and cancer are also being recognized (16–19), including in response to periodontal disease (20–23). This review will examine the current knowledge of the role polarization, or phenotypic changes, in macrophages and neutrophils is thought to play in the oral environment, especially during the development of periodontal disease.

## Macrophage Functions in the Oral Environment

Macrophages are located in the lamina propria below the epithelium and are among the first innate cells to interact with microorganisms and microbial products, and so are an important cell type under both homeostatic and disease conditions in the oral cavity. Under physiological conditions macrophages are important for cell turnover and maintenance of the extracellular milieu (24), while also being required to recognize, internalize and kill microbes in order to clear infections (25). The recognition of microbes by macrophages also results in production of proinflammatory cytokines, which contribute to inflammation initiation (26). Moreover, macrophages can act as antigen-presenting cells (APCs), collaborating with the early development of acquired immunity (27).

Under homeostatic conditions bone marrow derived monocytes enter tissue and differentiate into tissue specialized macrophages, including Langerhans cells (LCs), in the extracellular matrix of the mucosa (5, 28). Resident oral mucosal macrophages exhibit an array of diverse functions depending on different factors they encounter in their environment, including tissue architecture and microbiota (4, 29). A small portion of oral Langerhans cells that originate from monocytes are a more specialized subset of tissue resident macrophages (30, 31). Even within the same organ, macrophages can occupy different niches and have different specialized functions (28). Interestingly, recent research suggests that during tissue injury caused by myocardial infarction, stroke, and sepsis local proliferation of macrophages dominate over macrophage recruitment (32).

Oral macrophages are important for bridging the innate and adaptive immune response. Macrophages and oral Langerhans cells express high levels of MHCII and CD80/CD86, ingest particulate antigen and can present it to T cells (29, 31, 33, 34). Interestingly, oral LCs, unlike other tissue macrophages and similarly to dendritic cells, can migrate to lymph nodes to present antigen to T cells (30). Oral macrophages are also able to prime systemic immunity, as has been shown through systemic protection after sublingual vaccine administration and systemic antibody response to periodontal pathogens (4). In addition, when macrophages are depleted and mice infected intraorally with the periodontal pathogen *Porphomonas gingivalis*, specific antibody and cytokine responses are decreased, indicating the importance of macrophages in the adaptive immune response to oral bacteria (12).

## Macrophage Polarization in the Oral Environment

Macrophages can polarize to a variety of phenotypes ranging from alternatively polarized M2 macrophages to classically polarized M1 macrophages*in vitro*, which is well described in the literature (6, 28, 35, 36). In general, the M1 phenotype promotes a Th1 response and vigorous microbicidal and tumoricidal activity. In contrast, an M2 phenotype helps parasite clearance, dampens inflammation, promotes tissue remodeling, tumor progression, and possesses immune-regulatory functions (37). In reality, *in vivo* macrophages do not exist on dipoles as a population, but in a continuation of activation. The macrophage phenotype is plastic and in disease and in health there can be wide variety of multiple phenotypes present spanning from strict M1 to strict M2 and anywhere in between (38–40). Throughout this review *in vivo* macrophages will be referred to as M1-like or M2-like for this reason.

**Figure 1:**
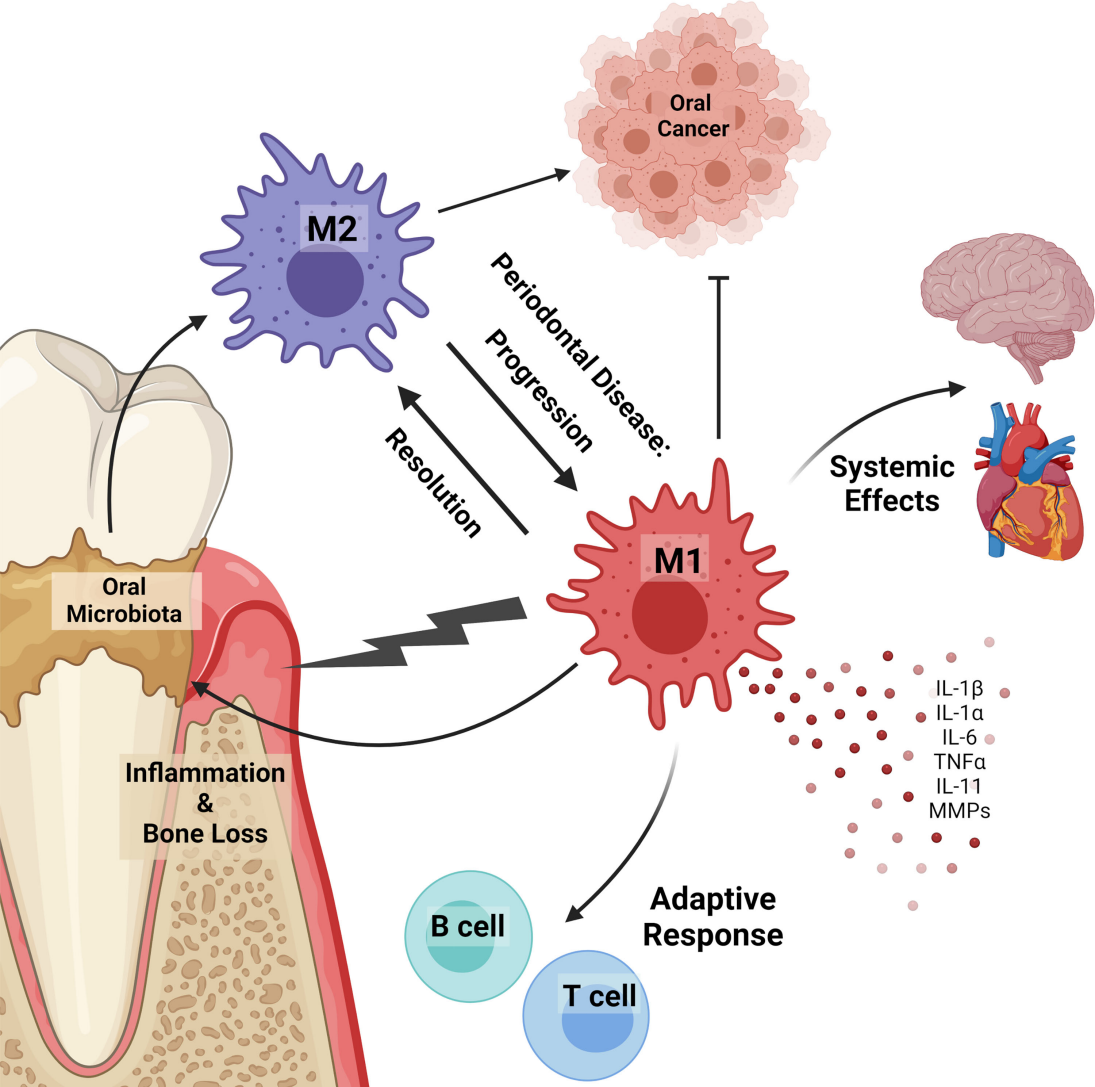
Macrophages in the oral cavity. Macrophages can polarize in response to oral microbiota and in disease. Inflammatory (M1) macrophages promote inflammation, alveolar bone loss, disease progression, microbiota dysbiosis, and prevent tumor development. Alternatively activated (M2) macrophages contribute to cancer progression and resolution of inflammatory diseases. Macrophages in the oral cavity are linked to systemic diseases, including those in the heart and brain. Inflammatory macrophages release pro-inflammatory cytokines (IL-1β, IL-1α, IL-6, TNFα, IL-11) and matrix metalloproteases (MMPs) that contribute to inflammation and alveolar bone loss. Under homeostatic and disease conditions oral macrophages work to bridge the innate and adaptive immune response by expressing antigens to B and T lymphocytes.

In the oral cavity, as with elsewhere in the body, predominate M1-like activation is generally associated with inflammatory diseases and predominate M2-like activation is associated with cancer (**Figure 1**) (6, 36). Indeed, dysregulation of the M1/M2 balance can lead to the progression of the inflammatory response and malignant oral diseases such as oral lichen planus and oral squamous cell carcinoma (SCC). M1-like macrophages can aid the progression of oral lichen planus and potentially induce malignant transformation. Conversely, M2-like macrophages [often termed tumor associated macrophages (TAMs)] aid SCC progression and favors an immunosuppressive tumor microenvironment (41). This is a general and well-known phenomenon of macrophages in many tumors, not just those in the oral cavity, with recent thorough reviews and so won’t be discussed further here (40, 42). Macrophage polarization can be driven by different bacterial species, microbial components, and host immune mediators (38, 43). For example, in murine macrophages a differential M1 or M2 profile occurs in response to representative Gram-negative or Gram-positive gastrointestinal bacteria, including probiotic strains, pathogens, commensals, and strains of food origin (44). In the oral environment, endotoxin and other bacterial products present in sterilized saliva polarize macrophages to an M1-like phenotype, with increased production of pro-inflammatory cytokines (45). Specific oral strains can also elicit unique responses, with data showing oral commensals generally elicit an M2-like phenotype while oral pathogens elicit a more M1-like phenotype (46). Furthermore, a switch from an predominantly M2-like phenotype to a predominantly M1-like phenotype is a potential mechanism for the advancement of periodontal disease (42). Studies using germ-free and specific pathogen free mice have also shed light on the ability of the oral microbiota to differentially modify phagocytes and their responses *in vivo*. For example, there is a significant decrease in IL-1β, an inflammatory cytokine mainly produced by macrophages, in germ free (GF) mice compare to specific pathogen free (SPF) mice (47), and oral Langerhans cells are significantly reduced in GF mice compared to SPF mice, but after microbial recolonization their numbers were restored (34).

## Macrophages in Periodontal Disease

Periodontal disease is a progressive inflammatory disease that results from dysbiosis in the microbiota and an overreactive immune response (48, 49). It is a complex disease, resulting from a myriad of different factors including genetics, environment, and microbes, with the microbial load from health to disease increasing from 10^2^ bacteria in health to 10^8^ bacteria in periodontitis (47). The importance of macrophages in the development and progression of periodontal disease has been shown through depletion of macrophages in a mouse model of periodontal disease, resulting in reduced *P. gingivalis* induced bone resorption (12). Although depletion of macrophages prevents bone resorption, it can also impairs bone regeneration (50). Data suggests aberrant expression of macrophage genes may affect their activation state and expression of signaling molecules, thereby contributing to disease progression (51). Indeed, many factors released by macrophages can be involved in periodontitis-associated alveolar bone loss. For example, inflammatory cytokines produced in high levels by macrophages including IL-1β, IL-1α, TNFα and IL-6 can activate osteoclasts making it likely that they play an important role in disease-induced bone resorption (26), and IL-1β is known to increase in human groups in association with an increased ratio of inflammatory macrophages over alternatively activated macrophages (52). In addition to cytokines, macrophages release a number of matrix metalloproteinases that are involved in degradation of the extracellular matrix and are important proteases involved in the progression of periodontal disease (53). Macrophage cytokines TNFα, IL-1, and IL-6 stimulate MMPs, all of which are expressed at higher levels in diseased periodontal tissue (54–56), with again some MMPs being linked to an increased M1/M2 ratio during disease (52). Together this shows the important role that macrophages and the myriad of factors they express can play in the destructive properties of periodontal disease.

As described above, macrophages can polarize to a broad range of phenotypes. On one end of this spectrum lays M1 macrophages, which generally have more inflammatory and microbicidal characteristics. Macrophages can polarize to an M1 phenotype through stimulation with IFNγ and TLR ligand interaction, as well as in response to periodontopathic bacteria. Macrophages stimulated with *P. gingivalis* or *P. gingivalis* LPS generally polarize to an M1-like inflammatory phenotype as shown by increased levels of pro-inflammatory cytokines and M1 specific surface markers (46, 57, 58). Mice infected with *P. gingivalis* also show increased levels of M1-like macrophages compared to M2-like macrophages (12). *Aggregetibacter actinomycetecometans*, another pathogen highly associated with periodontal disease, has also been shown to polarize macrophages to an M1-like phenotype (46, 59).

Periodontal disease is an inflammatory disease, so logically inflammatory macrophages (i.e., M1 macrophages) would infiltrate the periodontal tissue during disease: in recent years many studies have shown that this is indeed the case. A human experimental gingivitis study from Topoll and co-workers in 1989 was one of the first to show a decrease in anti-inflammatory macrophages and an increase in inflammatory macrophages (60). Now there have been multiple human studies showing increases of inflammatory M1-like macrophages in periodontal disease in comparison to healthy controls (42, 52, 61). These M1-like macrophages contribute to the inflammatory environment, promoting dysbiosis of the microbial community and periodontal disease progression (10). Macrophage interactions with normally commensal oral bacteria can also change, as seen with the increased, rather than decreased, survival of *Streptococcus gordonii* within IFNγ/LPS stimulated macrophages (62). Animal studies have also found that if the inflammatory response, especially by macrophages, is treated then progression of periodontal disease can be inhibited. One promising treatment is with the anti-inflammatory agent Resolvin-E1, which can resolve inflammation and regenerate bone and soft tissue to a healthy state (63). Such studies with anti-inflammatories have further illuminated periodontal disease as an inflammatory disease. Importantly, if alternatively activated, M2, macrophages are stimulated *in vivo* or injected into animal models of periodontal disease they promote healing and dampen osteoclast activity, thereby reducing bone resorption (50, 64, 65). On the other hand, *P. gingivalis* may promote the activation of macrophages into M2-like TAMs when combined with an OSCC microenvironment that can induce and promote OSCC growth (66).

## Systemic Effects of Oral Macrophages

There has been a recent increase in research focusing on the systemic implications of periodontal disease (48, 67–70). It is known that oral microbes can be found in extraoral locations after gaining access to the circulation, and periodontal disease-associated microbes have been found in multiple extra-oral sites including atherosclerotic plaques (71, 72) and the brain, showing they also can cross the blood-brain barrier (73, 74). Moreover, *P. gingivalis* appears capable of invading and converting myeloid-derived dendritic cells (mDCs) to an atherogenic phenotype in humans with chronic periodontitis (75). Along this line, the uptake of low-density lipoprotein (LDL) by transmigrated macrophages is enhanced in the presence of bacteria leading to accelerated foam cell formation and atherogenesis (48).

In addition to extra-oral bacteria affecting macrophages and systemic disease, the elevated systemic inflammation associated with periodontitis may have multiple systemic complications. For example, periodontal bacteria increase systemic IL-6 levels, driving the expansion of osteoclast precursors (OCPs) which can traffic to sites of bone resorption and differentiate into mature osteoclasts (76), suggesting that changes in the bone marrow may link periodontitis to other bone loss disorders, such as rheumatoid arthritis (77). Additionally, as individuals age there is increased inflammation in a nominally resting state and linked to this there is an increase in primarily M1 macrophage activation with age in nonhuman primates (78). Aging can enrich the gingival environment in anaerobic species leading to a dysregulated and persistent immunoinflammatory response (79). In this context, increases in prevalence and severity of periodontal disease have long been associated with aging (79). Recent evidence finds this long-associated age-related increase in periodontal disease is dependent, at least in part, on these age-related changes in macrophage activation towards an M1-like phenotype (11).

## Neutrophil Physiology

Neutrophils are the most abundant circulating leukocyte, which are among the first cells to respond to bacterial infections and pro-inflammatory signals. Neutrophils are produced in the bone marrow with 1 to 2×10^11^ neutrophils normally generated per day in an adult human (80). Mature neutrophils can be found in the bone marrow as the bone marrow reserve, circulating through the blood, or in tissues as resident neutrophils (81).

After production, mature neutrophils will remain in the bone marrow for about 5 days, forming the bone marrow reserve from which neutrophils can be rapidly mobilized in case of infection (82, 83). Once neutrophils cross the bone marrow sinusoidal endothelium (about 10^9^ neutrophils/kg body weight exit the bone marrow daily in humans) they enter the sinusoids and eventually migrate out into the general circulation (84, 85). Some of these neutrophils migrate into tissues, including through mucosal membranes and areas requiring constant immune surveillance, such as the oral cavity.

In human blood, neutrophils are generally believed to have a short half-life of about 8 hours, according to experiments in which neutrophils were labelled *ex vivo* and then evaluated *in vivo* (86). However, a study that labeled cells *in vivo via* deuterium-labelled water reported a surprising lifespan for circulatory neutrophils in humans of up to 5.4 days (87). Yet this paper’s methods received some criticism as orally administrated deuterium-labelled water would likely label bone marrow neutrophils as well as circulating neutrophils, therefore skewing their supposed longevity in blood (88). Another group proposes that the slow kinetics of labeled cells reported in Pillay 2010 may be due to the slow production of neutrophils in the bone marrow, rather than a long half-life in the blood (18, 89, 90).

These dynamic cells perform a wide range of protective functions, including chemotaxis toward stimuli, extravasation from vasculature and antimicrobial actions through phagocytosis, granule release, reactive oxygen species (ROS) production, and NETosis (91, 92). While neutrophils have traditionally been thought of as stable differentiated cells, evidence now demonstrates they are dynamic cells able to change their characteristics and behavior throughout their lifespan or in differing environments. Their potential responses can vary widely and change according to local signals released during acute or chronic inflammatory conditions, injury, infection, cancer, and autoimmunity. This plasticity has led to increasing interest in understanding their functional phenotypic heterogeneity, similar to other immune cell lineages (93).

## Neutrophils in the Oral Environment

**Figure 2:**
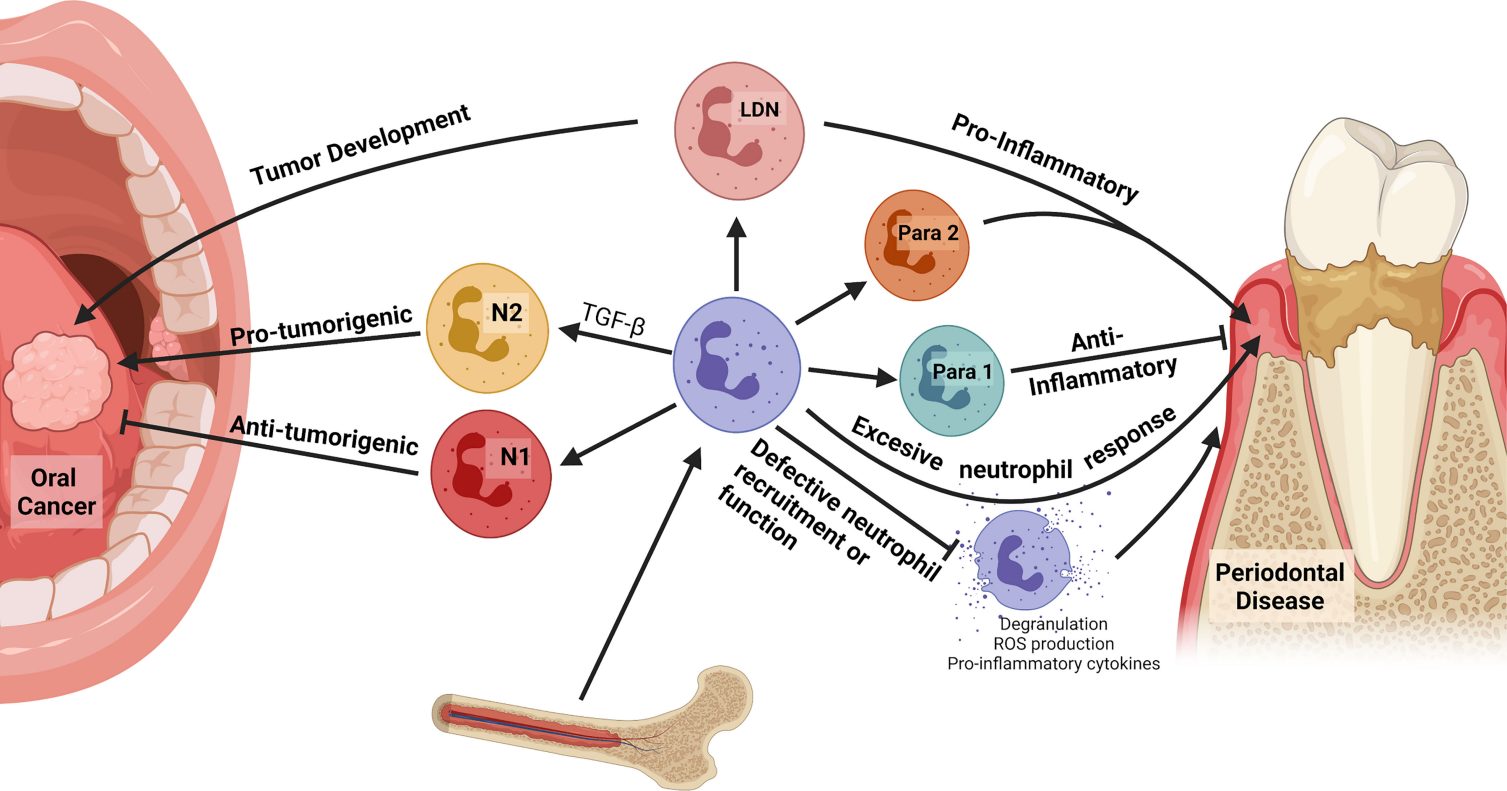
Neutrophils in the oral cavity. Neutrophils can polarize in response to oral microbiota and in disease, advancing or inhibiting disease progression. In oral cancers, N1 tumor-associated neutrophils (TANs) demonstrate anti-tumorigenic behaviors while N2 TANs are pro-tumorigenic, producing angiogenic factors and suppressing the antitumor immune response. Differentiation to the N1 or N2 phenotype is largely driven by TGF−β, which skews differentiation toward the N2 phenotype. Low-density neutrophils (LDNs) correlate with disease progression. They preferentially propagate in cancer and have a T-cell suppressive function, while proinflammatory LDNs are found in cases of autoimmunity. In addition, there is a subset of oral neutrophils that present with a significant increase in T cell receptor (TCR) expression and are recruited at high rates to sites of inflammation; however, their exact function is unknown. Increased neutrophil recruitment can contribute to the inflammation and alveolar bone loss characteristic of periodontal disease *via* production of ROS, pro-inflammatory cytokines, and degranulation. Furthermore, individuals with defective neutrophil recruitment or function due to genetic abnormalities are more susceptible to severe periodontal disease.

In the oral cavity, neutrophils have been identified in periodontal tissues, gingival crevicular fluid, and within dental biofilms (94–96). Neutrophils transmigrate through oral mucosa and comprise the majority of innate immune cells recruited to the gingival crevice, composing greater than 90% of the crevicular cells (97–101). Phenotypic differences between oral neutrophils in health and with location-specific differences are becoming better understood. Compared with circulating neutrophils, oral neutrophils present site-specific gene expression profiles in healthy individuals (102). Human studies have found that generally, oral tissue-resident neutrophils are in a later stage of their life cycle when compared to circulatory neutrophils and present a higher state of activation when compared to circulatory neutrophils, showing higher expression of activation markers CD11b, CD63 and CD66b, as well as higher constitutive ROS levels (103). In periodontal health a spectrum of neutrophil populations have been reported in humans, as characterized by two distinct subtypes of oral neutrophils; a ‘parainflammatory 1’ population which is similar to naïve blood neutrophils and a ‘parainflammatory 2’ population which is the more activated phenotype, possibly being in a primed state and may be crucial for response with the symbiotic biofilm of health (22). Another subset of oral neutrophils has also been identified that present with a significant increase in T cell receptor (TCR) expression compared with circulating neutrophils, and these cells show markedly increased recruitment to sites of inflammation. While the exact role of TCR expression in oral neutrophils is unknown, this supports a role for oral neutrophils in crosstalk between the innate and adaptive immune system in the oral cavity (**Figure 2**) (102).

## Neutrophils in Oral Cancer

Myeloid cells can promote tumor progression directly *via* immune suppression or indirectly *via* production of angiogenic factors, matrix-degrading enzymes, or growth factors. The most well characterized of these cells are TAMs that have properties of alternatively activated macrophages, or M2-like macrophages as described above (104). In recent years there has been increasing evidence showing phenotypic and functional plasticity in neutrophils, particularly in oral cancers (105). Like TAMs, these tumor-associated neutrophils (TANs) have differential states of activation/differentiation (104) TANs can polarize to anti-tumorigenic (N1) or pro-tumorigenic (N2) phenotypes. It is important to note that *in vivo* neutrophils, like macrophages, exist on a spectrum of activation and so in reality ‘N1’ and ‘N2’ are better understood as ends of a spectrum rather than discrete categories. *In vivo* animal studies show this change is largely directed by TGF−β, which skews differentiation toward the N2 phenotype. *In vitro* studies on human blood and tumor samples have found that N1 TANs express higher levels of immune activating cytokines and chemokines, lower levels of arginase, and have a heightened ability to kill tumor cells *in vitro*. N1 TANs express higher level of CCL3, ICAM-1, and TRAIL. N2 TANs support tumor growth by producing angiogenic factors and matrix-degrading enzymes and suppress the antitumor immune response. They are characterized by increased expression of gene markers, such as MMP9, VEGF-A, and BV8, as well as the decreased expression of CCL3, ICAM-1, and TRAIL (106, 107). Knowledge of TAN and circulating neutrophils in the context of head and neck cancer (HNC) of the oral cavity is somewhat limited, and even less is known about the oral neutrophils which populate the environment closely associated with evolving HNC. These oral neutrophils represent a unique population with functional and phenotypic proprieties distinct from other compartments such as the tumor or mucosal tissue (108). Localization of the oral neutrophils and neutrophil-derived products along with the tumor-derived microenvironment rich is growth factors and cytokines may influence tumor development and differential neutrophil plasticity. For example, oral neutrophils from patients with untreated oral cancer demonstrate distinct functional properties with lower chemotactic ability, superoxide production and reduced killing of microbes (109). Understanding phenotypic differences and properties of oral neutrophils in oral cancer provides a relevant measure of local response in the tumor environment with high prognostic value.

Neutrophil granule changes have also been demonstrated to reflect differential phenotypes as during some inflammatory states or other disease conditions, there exists a subset population of neutrophils which co-sediment with peripheral blood mononuclear cells (PBMCs) due to their unique granularity compared to normal mature neutrophils known as low-density neutrophils (LDNs) (110). LDN numbers correlate with pathological conditions such as rheumatoid arthritis, lupus and cancers including potentially in the early phase of oral squamous cell carcinoma development (111–113). Their overall function is dependent on inflammatory stimuli. For example, a proinflammatory LDN phenotype has been described *in vivo* animal studies for LDNs in autoimmunity and infection while in cancer LDNs primarily have a T cell suppressive function and are often referred to as myeloid-derived suppressor cells (111, 114).

## Neutrophils in Periodontal Disease and Inflammation

The association between periodontal disease and neutrophil presence is well established. The human oral cavity has a constant bacterial presence that is kept under control by equally constant immune surveillance. Neutrophils comprise the majority of innate immune cells recruited to the gingival crevice and tissue to maintain physiological health, while documented increases in the number of oral neutrophils during periodontal disease which correlates with clinical severity in response to the dysbiotic biofilm and inflammatory changes (98, 101, 102, 115). A lack of neutrophil infiltration into the oral cavity or defects of neutrophil function lead to an increase in periodontal disease (9). Congenital defects in neutrophil development or egress from the bone marrow resulting in significant neutropenia are linked to severe periodontal disease (116). Likewise, defective extravasation of neutrophils into tissue and impaired immune response are also linked to increased periodontal disease. For example, Leukocyte adhesion deficiency are a group of congenital disorders in which neutrophils have defective expression or function of adhesion molecules involved in attachment and migration through the vascular endothelium, resulting in neutrophilia with few neutrophils in peripheral tissues leading to subsequent recurrent infections including severe periodontal disease (117). On the other hand, increased neutrophil recruitment in attempt to control the dysbiotic biofilm characteristic of periodontal disease or dysregulation of appropriate trafficking and resolution of the neutrophil response contributes to much of the tissue damage in periodontal disease (10). Many of the oral microbes abundant in the dysbiotic community of periodontal disease are able to disarm or impair local neutrophil responses, also rendering these cells ineffective in the gingival tissues (118, 119).

A variety of studies have suggested that neutrophils during periodontal disease are heterogenous populations which likely respond to local microenvironment and microbe cues. Classical studies have revealed that circulating neutrophils from patients with periodontal disease have impaired chemotactic ability, responsiveness and directionality along with changes in surface expression of CD11b or CD16 (120–122), which suggest potential of functional neutrophil subsets. Recent detailed comparative analysis of oral neutrophil surface markers has further revealed distinct upregulation of specific markers during periodontitis characterized by a pro-inflammatory signature along with a functionally activated phenotype with elevated degranulation, phagocytosis, ROS production and NET formation (22, 103, 123). Studies on blood and oral rinses from healthy and chronic periodontitis patients have revealed differential expression of neutrophil surface markers in different biological compartments (124) and during transmigration (125), however ongoing studies are required to identify definitively if observed molecular changes and phenotypic features in oral neutrophils during disease represent active subset switching in response to stimuli or a true differentiated population.

Despite appropriate clinical therapy, a subset of periodontitis patients do not respond effectively and present with continuing disease progression and clinical attachment loss that does not correlate with plaque levels, microbiology assessments, and treatment compliance. Such patients are diagnosed with refractory periodontitis (RP) (126). A unique hyperactive oral neutrophil phenotype characterized by increased potential for ROS production has been identified in these RP patients. These cells were found to produce ROS at a level approaching the maximal capability of the cells (127). While appropriate ROS production is crucial for effective bacterial killing, excessive ROS can contribute to periodontal attachment loss by damaging the extracellular connective tissue (128).

While characteristic neutrophil surface marker signatures have been demonstrated, a distinct neutrophil-specific marker has been reported to be associated with periodontal disease. CD177, also called neutrophil antigen B1 (NB1) or human neutrophil antigen 2a (HNA-2a), is a glycosylphosphatidylinositol (GPI)-linked glycoprotein expressed on the plasma membrane and in granule membranes of neutrophils (129). A high proportion of CD177-expressing neutrophils have also been found in the gingival crevicular fluid (GCF) of periodontitis patients. The proportion of CD177-expressing neutrophils in circulation varies between individuals with a relatively stable bimodal, or occasionally trimodal, expression pattern (130, 131). In humans, the proportion of CD177-expressing neutrophils ranges from 0% and 100% of circulating neutrophils. CD177 can interact with PECAM-1 (expressed on endothelial cells, platelets, monocytes, and granulocytes), which is a key player in neutrophil migration from bloodstream to tissue (132). The proportions of CD177^+^ neutrophils is higher in GCF from periodontitis patients, as compared to blood from the same donor and this accumulation of CD177^+^ neutrophils in inflammatory exudate was not seen in two different models of aseptic inflammation, suggesting that this is a periodontitis specific phenotype. Furthermore, the CD177^+^ neutrophil subtype does not accumulate in the GCF of healthy subjects (133).

As periodontal disease is considered an inflammatory condition with systemic associations, neutrophil phenotypes related to inflammation are likely relevant to health in the oral cavity as well as systemically at distant sites. Recent work has demonstrated that oral inflammation occurring during human experimental gingivitis or ligature induced models of murine periodontitis has systemic effects to produce an exacerbated immune response at a secondary site during a secondary insult (134). This supports a larger role of oral inflammation and particularly neutrophil-derived changes broadly throughout the body.

## Summary

There is an abundance of work highlighting the importance of phagocytic cells, including macrophages and neutrophils, under conditions of health and disease in the oral environment. Both of these cell types play specialized roles in part by polarizing to a variety of phenotypes to alter their phenotypic responses in health and disease. As described in this review, a key role for such polarization in the context of oral diseases, including periodontal disease and oral cancer is well documented. However, while there is increased understanding of the roles for innate immune cell polarization in the oral environment in recent years, their remains a lack of insight into molecular mechanisms fueling the responses of these phagocytes. Future characterization of the plasticity of innate immune cells will provide important information to decipher their detailed roles in driving pathogenic conditions in the oral cavity.

## Author Contributions

SM, NA, AE, MV, and JK all contributed to the writing and editing of the manuscript. All authors contributed to the article and approved the submitted version.

## Funding

The authors received funding from the following, NIH grants F31DE029400 (SM), F31DE030705 (NA), R01DE027073 (MV), R01DE028307 (JK) and T32DE023526, as well as Fondo Nacional de Ciencia y Tecnología FONDECYT 1180666 (AE).

## Conflict of Interest

The authors declare that the research was conducted in the absence of any commercial or financial relationships that could be construed as a potential conflict of interest.

## Publisher’s Note

All claims expressed in this article are solely those of the authors and do not necessarily represent those of their affiliated organizations, or those of the publisher, the editors and the reviewers. Any product that may be evaluated in this article, or claim that may be made by its manufacturer, is not guaranteed or endorsed by the publisher.
